# Maternal diabetes mellitus as an independent risk factor for clinically significant retinopathy of prematurity severity in neonates less than 1500g

**DOI:** 10.1371/journal.pone.0236639

**Published:** 2020-08-03

**Authors:** Chibuzor Nonye Opara, Mopelola Akintorin, Allison Byrd, Natascha Cirignani, Similolu Akintorin, Kenneth Soyemi

**Affiliations:** 1 Department of Pediatrics, John H Stroger Hospital of Cook County, Chicago, Illinois, United States of America; 2 A. T. Still University School of Osteopathic Medicine, Arizona, United States of America; 3 Keck School of Medicine University of Southern California, Los Angeles, California, United States of America; University of Florida, UNITED STATES

## Abstract

**Background:**

Retinopathy of prematurity (ROP) is a significant morbidity in preterm babies. Multiple risk factors for severe ROP have been extensively studied, however, only a few studies have included maternal diabetes mellitus (MDM) in their assessment. ROP and diabetic retinopathy are both retinal vascular diseases in which there is leakage and/or neovascularization from damaged retinal vessels. Diabetes may affect ROP development; however, there are conflicting results on the association between MDM and ROP.

**Objective:**

To determine if MDM is an independent risk factor for clinically significant ROP (ROP > Stage II) in neonates weighing less than 1500g.

**Design/Method:**

We conducted a retrospective cohort study of neonates weighing <1500g who were delivered or transferred into our institution from 2007 through 2017. Logistic regression was used to analyze the association between severe ROP and MDM. The risks for the different stages of ROP from MDM were compared using chi-square linear trend test.

**Results:**

We extracted 883 paired maternal-neonatal data. The mean (standard deviation) gestational age and birthweight were 28.5 (2.9) weeks and 1052.7 (300.9) grams, respectively. Of the 883 mothers, 72 (8.2%) had DM. The incidence of ROP and severe ROP was 42.4% (374/883) and 6.5% (57/883) respectively. The odds ratio comparing MDM and severe ROP was 3.47 [95% CI: 1.51–7.96]; p<0.01). Compared to Stage I, the risk of MDM in infants with ROP increased from 1.49 in Stage II ROP to 2.59 in Stages III&IV. Severe ROP was associated with infant steroid use (OR: 5.92 [95% CI: 2.83–12.38]; p <0.01), sepsis (OR: 2.13 [95% CI: 1.09–4.14]; p = 0.03) chorioamnionitis (OR: 1.90 [95% CI: 1.03–3.50]; p = 0.04), and maternal steroid use (OR: 0.51 [95% CI: 0.32–0.79]; p<0.01).

**Conclusion:**

Maternal diabetes is associated with ROP and the strength of association increased with increasing severity of ROP.

## Introduction

Retinopathy of prematurity (ROP), formerly known as retrolental fibroplasia, represents a significant morbidity in the preterm babies as it remains one of the important causes of blindness nationally and globally. The incidence of ROP and severe ROP varies from one geographic region to another and increases with reducing gestational age (GA) and birth weight (BW) [1]. A recent multicenter study of United States and Canadian hospitals found ROP in 43.1% of newborns with BW ≤1500g [2], while another multicenter study in the US found ROP in 68% of newborns with BW <1250g [3]. One population-based study noted an increase in the incidence of severe ROP from 3% to 34% as GA decreased from 27 to 24 weeks [4]. Overall, most patients with ROP undergo spontaneous resolution as less than 10% would eventually require treatment [5].

Some authors have described the public health burden of ROP in “epidemics” with the “first and second epidemics” historically observed in industrialized nations [6]. More recently, the emergence of a “third epidemic” mostly in middle- and low-income countries is of concern [6]. Of note, the “first epidemic” was observed in the 1940s and 1950s among premature babies in the United States and Western Europe with uncontrolled use of supplemental oxygen as the primary risk factor. The “second epidemic” was observed in the 1970s as a result of increased survival rates of extremely premature babies who incidentally experienced more cases of acute severe ROP compared to the larger neonates. This consequently provided more evidence that low birth weight and prematurity are primary risk factors for ROP. It also formed the basis of the recommendations by relevant bodies including the American Academy of Pediatrics (AAP) and American Academy of Ophthalmologists (AAO) to screen for ROP in all babies with BW ≤1500g or GA ≤30 weeks, as well as selected neonates with BW between 1500 and 2000g or GA >30 weeks believed by their attending pediatrician or neonatologist to be at high risk for ROP [7].

On the other hand, the recent development in the emerging economies have been attributed to their higher rates of premature births, as well as babies with severe disease requiring treatment. This points to higher rates of neonatal exposure to the risk factors of ROP in these countries which are mostly being controlled in industrialized nations.

Terry in 1942 was the first to describe ROP and specify its association with prematurity [8]. Since then, several risk factors have been studied and some noted to be associated with ROP. While most authors agree that birthweight/gestational age and oxygen use play a role, some have suggested that maternal diabetes mellitus (MDM) or hyperglycemia may be implicated in ROP as well [9–11]. This is not conclusive as some researchers have found no association between maternal diabetes or hyperglycemia and ROP [12–14].

Some commonalities exist between ROP and diabetic retinopathy based on pathogenesis, as both are retinal vascular diseases, in which there is leakage and/or neovascularization from damaged retinal vessels based on retinal ischemia. Moreover, diabetes is a risk factor for preterm delivery; and prematurity is the leading cause of neonatal morbidity including development of ROP. This suggests that maternal diabetes mellitus may have both direct and indirect impact on ROP development. The objective of this study is to determine if MDM is an independent risk factor for clinically significant ROP (ROP > Stage II) in neonates weighing less than or equal to 1500g.

## Methods

We conducted a retrospective cohort study to evaluate the association between severe ROP and MDM, while adjusting for multiple risk factors. The study was approved following an expedited review by the Cook County Health and Hospital System Institutional Review Board (IRB), Study ID 18-169X. Prior to accessing the medical records, we obtained a waiver for the requirement of informed consent from the IRB. The IRB approval was obtained on November 26, 2018. We subsequently started accessing patient data on November 27, 2018. All research activities were conducted in accordance with the Declaration of Helsinki.

### Data collection

The study population included all infants with birth weight ≤1500g delivered at, or transferred to, our study institution, John H Stroger Hospital of Cook County, Chicago, Illinois. The study period was from January 2007 through December 2017. We reviewed data on mothers and infant pairs from their electronic medical records. Records with incomplete charting of infant/mother pairing were excluded. Equally excluded were infants who died prior to discharge, were transferred to another hospital prior to eye exam, discharged prior to initial comprehensive eye exam and did not follow up at our center.

**Fig 1:**
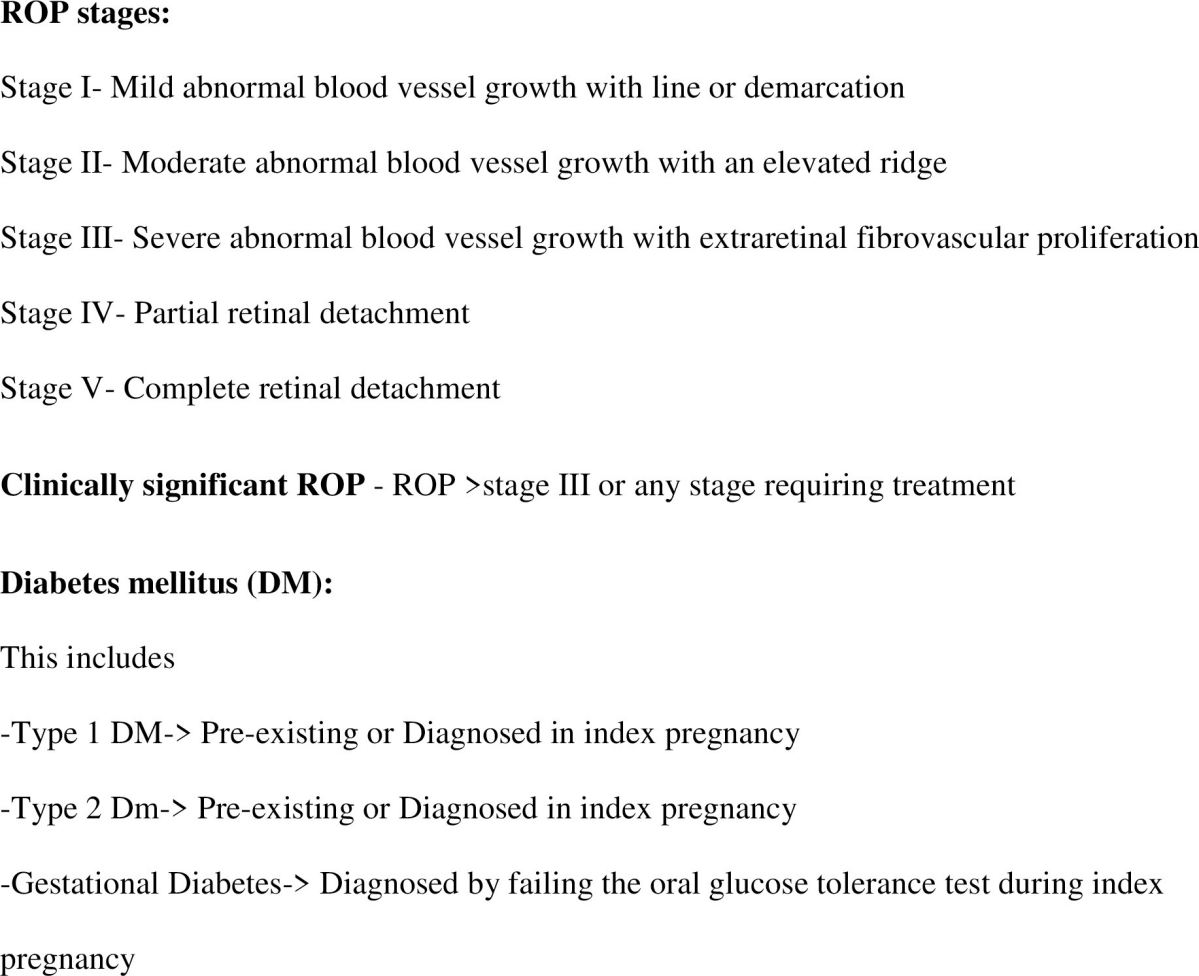
Classification of ROP and definition of terms.

The procedure and schedule for screening for ROP was done by attending pediatric ophthalmologists on staff in accordance with the standard recommendations by the AAP, AAO, the American Association for Pediatric Ophthalmology and Strabismus (AAPOS), and the American Association of Certified Orthoptists (AACO) (7). The classification and severity of ROP was documented based on approach stipulated by the International Classification for Retinopathy of Prematurity (ICROP) as shown in Fig 1 [15].

For our purposes, severe ROP, or clinically significant ROP, represented ROP ≥ Stage III or any stage requiring treatment. In addition, it is noteworthy that none of infants with Stage I or Stage II ROP had plus disease or required treatment; hence, severe ROP in our study only included those with greater than or equal to Stage III disease. Maternal diabetes mellitus encompassed gestational diabetes and pre-gestational diabetes, including Type I or Type II DM which was pre-existing or diagnosed during the index pregnancy. Covariates and potential confounders analyzed include birthweight, use of mechanical ventilation, antibiotics, or steroids in the neonates, as well as presence of bronchopulmonary dysplasia (BPD), intraventricular hemorrhage (IVH), sepsis, patent ductus arteriosus (PDA), or respiratory distress syndrome (RDS). Some maternal factors included were maternal age, ethnicity, and presence of chorioamnionitis.

### Statistical analysis

**Fig 2:**
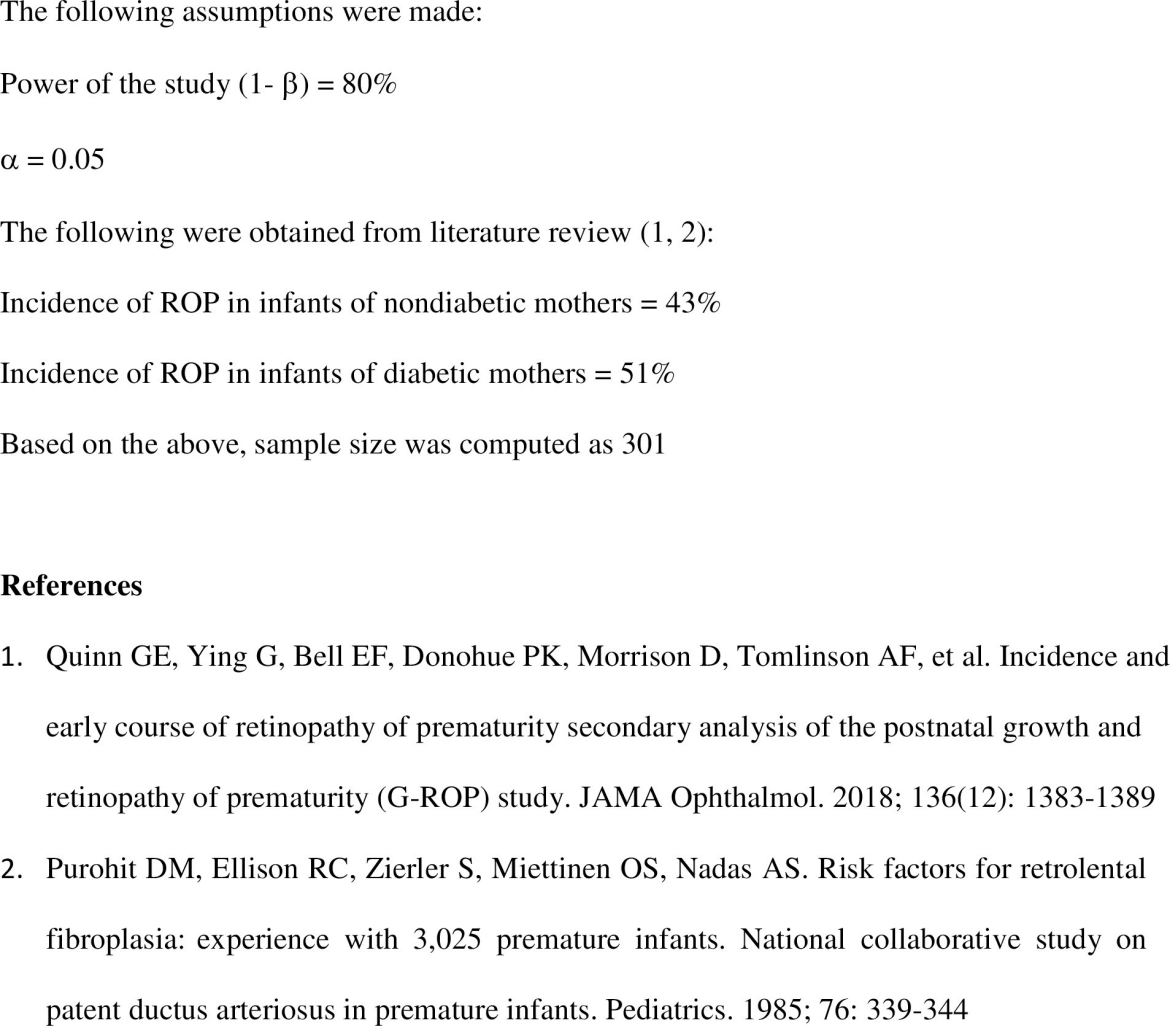
Sample size calculation.

We used a standard data collection form to extract clinical data into a database from the electronic medical records. Double entry method was utilized in order to minimize errors by further extrapolating the data from the original database into Microsoft Excel. Data analyses were carried out using Epi info Version 7 (Centers for Disease Control and Prevention, Atlanta, GA) and IBM PASW (SPSS, Chicago, IL). Descriptive statistics were represented using mean and standard deviation for continuous variables; and frequency (percentage) for categorical variables. The Student T-test was used to compare means of continuous variables including diabetes vs non- diabetes mothers. For categorical variables, chi-square independence test was used for comparison of independent groups. Sample size was calculated using pertinent information including α level of 0.05 and power of 0.80 as shown in Fig 2. A chi-square linear trend test was done to compare the risk of the different stages of ROP for diabetes vs non-Diabetes exposure using stage 1as the reference group. Logistic regression analysis was completed to evaluate the relationship between severe ROP and maternal DM as well as other pertinent maternal and neonatal factors. Odds ratios and their 95% Confidence intervals were computed, and p<0.05 was considered statistically significant for univariate and multivariate analyses.

## Results

**Fig 3:**
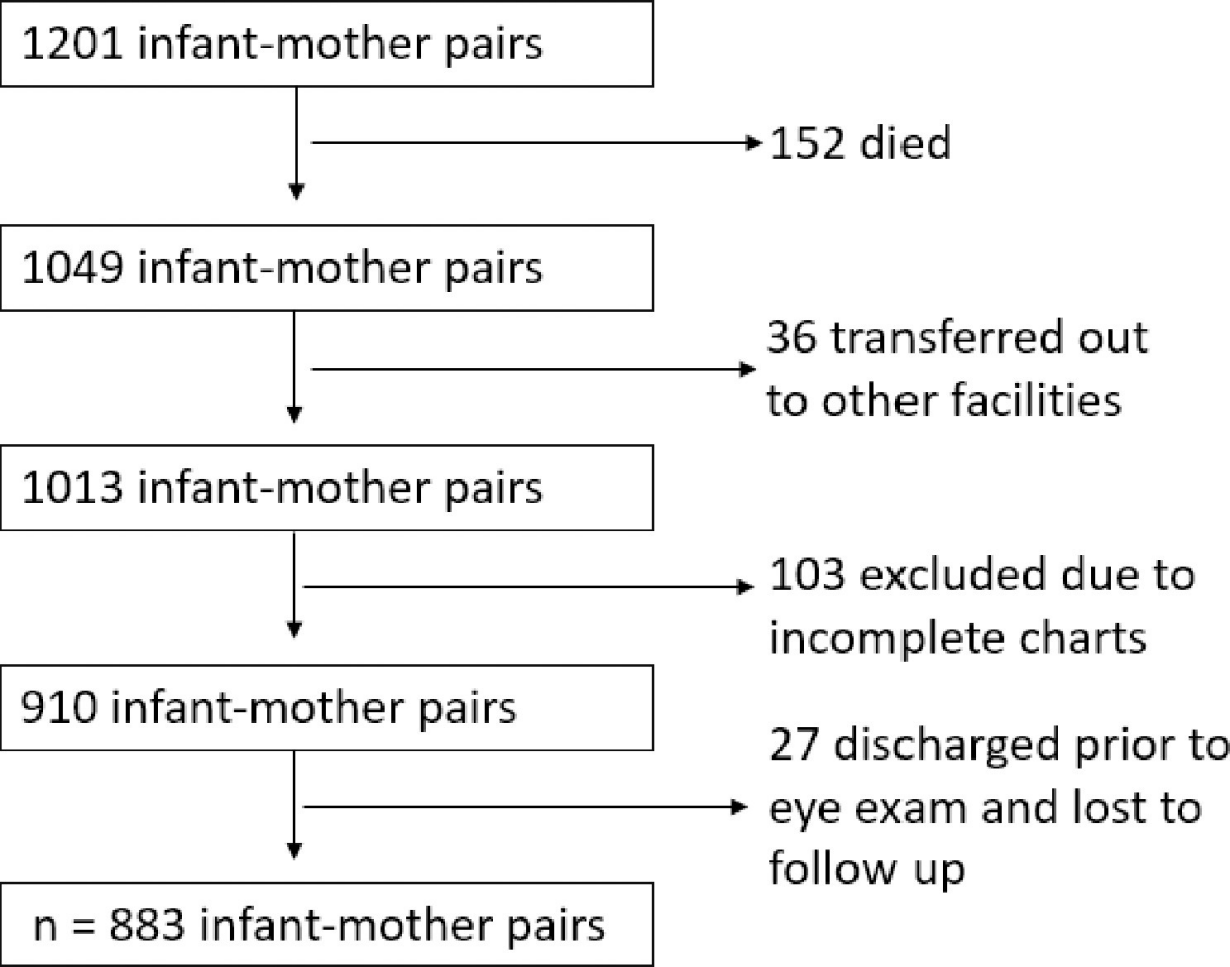
Flow chart of exclusion criteria.

There were a total of 1201 infants with birth weight ≤1500g delivered at, or transferred to, our hospital during the study period as shown in Fig 3. Of these, 152 were deceased and another 36 infants transferred out of the hospital to other facilities for escalation of care and other different reasons. Twenty-seven infants were excluded because they were discharged prior to having their eye exams done and did not follow up at our hospital. Incomplete medical charts were noted in the electronic medical records of 103 infants and were subsequently excluded. Therefore, a total of 883 infant-mother pairs were included in the study for analysis. It is noteworthy that based on our sample size calculation, 301 infant-mother pairs were required for the study (Fig 2).

As regards race and ethnicity of the 883 mothers, 189 (21.4%) were Non-Hispanic Whites (NHW), 589 (66.7%) were Non-Hispanic Blacks (NHB), while other races accounted for 105 (11.9%). Overall mean (SD) age in years for all participants was 26.8 (7.0) years; with the following mean (SD) ages for different races: NHW, 28.1 (6.9) years; NHB, 26.4 (6.8) years; and Other races 26.8 (7.5) years. The mean (SD) gestational age and birthweight of all infants in the study were 28.5 (2.9) weeks and 1052.7 (300.9) grams, respectively.

The incidence of ROP in our study population was 42.4 percent, representing 374 of all the infants. Of these, 201 infants (53.7%) had Stage II disease, while 116 infants (31%) had Stage I disease, respectively. There is no clear reason behind the uncommon finding of a higher incidence of Stage II ROP compared to Stage I in our study population. One probable explanation could be that higher stages of ROP occur more commonly in babies who are more premature and those with lower birth weights. Our study population characteristics in terms of gestational age and birth weight appeared to be closer to this lower end of the spectrum.

As previously noted, none of the infants with Stage I or Stage II ROP in the study population had plus disease or required treatment; hence, severe ROP in our study encompassed only infants with ≥Stage III disease. Consequently, the incidence of severe ROP, or clinically significant ROP, in our study population was 6.4%, which also represents 15.3% of all infants with ROP in the study. Of note, infants with severe ROP had Stage III and Stage IV disease, as no infant in our study population had Stage V disease.

The demographic characteristics of the ROP group vs non-ROP group were outlined in Table 1. Overall, birthweight and gestational age were significantly lower; while number of ventilation days, hospital length of stay (HLOS), and number of oxygen days, were significantly higher in the ROP group vs the non-ROP group, respectively.

**Table 1:** Clinical differences between ROP group and non-ROP group.

Variable	ROP Group (n = 374) Mean (SD)	Non-ROP Group (n = 507) Mean (SD)	Mean Difference	95% CI Mean Difference	P^a^
Gestational Age (weeks)	26.6 (2.4)	29.9 (2.4)	3.4	3.0 to 3.7	<0.0001
Birth Weight (g)	862.8 (242.4)	1191.6 (261.2)	328.8	294.9 to 362.7	<0.0001
Length of Stay (days)	93.8 (39.0)	50.7 (26.3)	-43.0	-47.4 to -38.7	<0.0001
Number of Ventilator Days	31.9 (27.8)	9.7 (14.3)	-22.1	-25.4 to -18.8	<0.0001
Number of Oxygen Days	71.5 (44.5)	24.9 (28.2)	-46.6	-51.7 to -41.4	<0.0001

^a^ T-test.

Of the 883 mothers in the study, 72 (8.2%) had diabetes mellitus, with gestational diabetes accounting for 32 of the 72 mothers with diabetes (44.4%). There were 13 mothers with Type 1 diabetes (18.1%) and 27 mothers with Type 2 diabetes (37.5%), respectively. A total of 10 out of the 72 infants of diabetic mothers, or 13.9 percent, had severe ROP. The demographic characteristics of the diabetic vs non-diabetic maternal infant pairs were outlined in Table 2. Overall, the two groups were comparable with respect to most of the demographic characteristics including birthweight, gestational age, number of ventilation days, and number of oxygen days. However, there was a statistically significant difference between the two groups in maternal age.

**Table 2:** Clinical differences between diabetes group and non-diabetes group.

Variable	Diabetes+Mean (SD)	Diabetes-Mean (SD)	Mean Difference	95% CI	P^a^
Birth Weight (g)	1099.9 (281.7)	1048.5 (302.4)	-51.4	-123.9 to 21.2	0.1651
Gestational Age (weeks)	29.1 (3.2)	28.5 (2.9)	-0.6	-1.3 to 0.1	0.0870
Maternal Age (years)	**29.2 (5.9)**	**26.6 (7.0)**	**-2.6**	**-4.3 to -0.9**	**0.0021**
Number of Ventilation Days	23.7 (21.6)	20.7 (25.0)	-2.9	-10.1 to 4.1	0.4098
Number of Steroid Doses	22.6 (18.4)	26.6 (30.4)	4.0	-11.2 to 19.2	0.6006
Number of Oxygen Days	45.8 (40.5)	46.8 (43.8)	1.0	-10.3 to 12.3	0.8569

^a^ T-test.

Using unadjusted logistic regression analysis, the risk of ROP associated with maternal diabetes was 2.64 (95% CI: 1.26–5.44) (p<0.01). Neonatal factors included in the multivariate analysis model comprised birthweight, use of antibiotics or steroids in the neonates, as well as presence of necrotizing enterocolitis (NEC), intraventricular hemorrhage (IVH), sepsis, patent ductus arteriosus (PDA), or respiratory distress syndrome (RDS). Some maternal factors included were maternal age, maternal diabetes mellitus, ethnicity, prenatal care, maternal steroid use, and presence of chorioamnionitis.

Data from the multivariate logistic regression are shown in Table 3. The results of the multivariate analysis showed that there was a statistically significant positive association between maternal diabetes and severe ROP. Essentially, the odds of an infant with severe ROP having a diabetic mother is 3.5 times higher compared to a non-diabetic mother (OR: 3.47 [95% CI: 1.51–7.96]; p<0.01). Other statistically significant independent risk factors for severe ROP in the study included infant steroid use, sepsis, maternal steroid use, and maternal chorioamnionitis.

**Table 3:** Multivariate logistic regression analysis.

Variable	Odds Ratio	95% CI	Coefficient	SE	Z Statistic	P value
**Maternal Chorioamnionitis**	**1.9038**	**1.0344–3.504**	**0.6439**	**0.3112**	**2.0686**	**0.0386**
Maternal Age	0.9794	0.9504–1.0093	-0.0208	0.0153	-1.3568	0.1748
Maternal Hypertension	0.6925	0.4231–1.1335	-0.3674	0.2514	-1.4616	0.1438
Prenatal Care	0.8002	0.4762–1.3447	-0.2229	0.2648	-0.8416	0.4
**Maternal Steroids**	**0.5059**	**0.3245–0.7888**	**-0.6813**	**0.2266**	**-3.0066**	**0.0026**
**Maternal DM**	**3.4687**	**1.5122–7.9567**	**1.2438**	**0.4236**	**2.9632**	**0.0033**
**Neonatal Steroids**	**5.9229**	**2.8326–12.3846**	**1.7788**	**0.3763**	**4.7265**	**0**
NEC	0.9187	0.3625–2.3281	-0.0848	0.4744	-0.1788	0.8581
RDS	3.3198	0.4214–26.1547	1.1999	1.0531	1.1394	0.2545
Gestational Age	0.8292	0.6792–1.0122	-0.1873	0.1018	-1.8405	0.0657
Birth Weight	0.9985	0.9966–1.0005	-0.0015	0.0010	-1.5050	0.1323
BPD	2.3199	0.8192–6.5692	0.8415	0.5311	1.5846	0.1131
IVH	1.5889	0.7991–3.1594	0.4630	0.3507	1.3203	0.1867
**Neonatal Sepsis**	**2.1289**	**1.0948–4.1394**	**0.7556**	**0.3393**	**2.2270**	**0.0259**

BPD, bronchopulmonary dysplasia; DM, diabetes mellitus; IVH; intraventricular hemorrhage; NEC, necrotizing enterocolitis; RDS, respiratory distress syndrome.

The data from the chi-square linear trend test used to evaluate for the probability of a sequential association between the different stages of ROP and maternal diabetes were presented in Table 4. The table shows that the overall incidence of maternal diabetes among all infants with ROP was 10.2%. The proportion of infants with ROP Stage I and Stage II that had diabetic mothers were 6.9% and 10%, respectively. The incidence of maternal diabetes among infants with severe ROP was found to be 17.5%. Furthermore, the chi-square linear trend test showed that the odds of maternal diabetes being present in an infant with ROP increased from 1.49 in Stage II ROP to 2.59 in Stages III&IV ROP (severe ROP) compared to the reference group, represented as the infants with Stage I ROP.

**Table 4:** Chi square linear trend test of DM Vs ROP severity.

ROP Stage	DM +	DM -	Total	Odds of Exposure	Odds Ratio
1^a^	8	108	116	0.07	1
2	20	181	201	0.11	1.49
3 and 4	10	47	57	0.21	2.59
Total	38	336	374	Extended Mantel-Haenszel Chi Square for linear trend = 3.75; p-value (1 degree of freedom) = 0.05284	

^a^ Reference group.

## Discussion

This research study has some findings of clinical significance: (1) Maternal diabetes mellitus was found to be an independent risk factor for severe ROP, or clinically significant ROP (ROP Stage III or higher) in neonates weighing less than or equal to 1500g, after adjusting for multiple relevant covariates. (2) The strength of the association between ROP and maternal diabetes increased as the ROP stages became more advanced.

### Comparison with other studies

Overall, our study showed that maternal diabetes increased the risk of an infant developing severe ROP by 3.5 times. Several authors have assessed a number of risk factors for severe ROP including birthweight, GA, sepsis, RDS, BPD, PDA, neonatal hyperglycemia, blood transfusion, supplemental oxygen administration, mechanical ventilation, and preeclampsia [4, 16–21]. Based on extensive literature search, ours is one of a handful of studies that included maternal diabetes in the assessment of the risk factors for severe ROP [9, 22–24]. Tunay and colleagues studied the relationship between maternal diabetes and ROP in infants with birthweight >1500g and found 25-fold and 6-fold increase in the risk of ROP and Type 1 ROP, respectively, among infants of diabetic mothers [9]. The strength of association in this study was higher in ROP vs severe ROP as opposed to our finding where we noted a sequential increase in strength of association with increasing severity of ROP. The fact that their research was done on babies with BW > 1500g as against infants with BW ≤1500g in our study could explain the difference.

The incidence of severe ROP has been found to be higher among smaller babies although studies have shown that maternal diabetes is associated with both low birthweight and high birthweight [16, 25]. The National Collaborative Trial on Patent Ductus Arteriosus studied infants with birthweights < 1750g and observed 8.5% increase in the rate of ROP among infants of diabetic mothers compared to babies born to nondiabetic mothers [26]. They did not evaluate the relationship between maternal diabetes and severe ROP.

Furthermore, there is a growing body of research signifying an association between neonatal hyperglycemia and severe ROP, as well as ROP of all stages [10, 11, 27]. Kaempf and colleagues found that both the number and severity of hyperglycemic episodes as well as a higher mean blood glucose were associated with both mild and severe ROP [27]. Garg and colleagues reported that the risk of severe ROP in infants with BW <1000g increased 2.7-fold for every 10mg/dL rise in mean serum glucose during the neonatal period [10]. These findings lend support to our results showing an association between severe ROP and maternal diabetes. This is more so because it is plausible that infants of diabetic mothers in our study were exposed to some levels of hyperglycemia in utero, given the retrospective nature of our study.

Conversely, Rehan and associates carried out a double cohort study on very low birthweight infants (BW < 1500g) in Canada and found no association between severe ROP or ROP of any stage and maternal diabetes [28]. An Israeli-based prospective study of very low birthweight preterm infants with GA 24 to 33 weeks concluded that infants of diabetic mothers did not have an increased risk of developing ROP stages III and IV [14]. The variability in the study population baseline characteristics, study design, study period, and glycemic control could explain the differences in the findings of these studies [16].

Other significant findings include the incidence of maternal diabetes in our study population noted to be 8.1%. The incidence of ROP was 42.3% while the incidence of severe ROP was 6.3%, respectively. These findings are comparable to those reported in similar reputable research studies [2, 9, 22]. On the other hand, two of the largest previous studies (Early Treatment for Retinopathy of Prematurity [ETROP] Study and Cryotherapy for Retinopathy of Prematurity [CRYO-ROP]) reported significantly higher rates of ROP and severe ROP [29, 30]. However, it is noteworthy that their study populations were infants with BW < 1251g.

Our multiple logistic regression analysis demonstrated that neonatal sepsis, maternal chorioamnionitis and use of steroids in infants were independent risk factors for severe ROP. Use of steroids in mothers during pregnancy was significantly associated with reduced risk of severe ROP. Several studies have identified neonatal sepsis, neonatal steroid use and maternal steroid use as well as maternal chorioamnionitis as independent risk factors for severe ROP [9, 22, 31–33]. These risk factors have been associated with increased length of stay, prolonged morbidity, and most especially, increased ventilator days. Increased ventilator days, by extrapolation, points to greater exposure to supplemental oxygen which has been shown to be a primary risk factor for developing ROP.

### Strengths and limitations

Our study demonstrates a positive association between maternal diabetes and severe ROP in very low birthweight preterm infants. We consider our focus on severe ROP a strength of the study because it stems from the recognition of the need to judiciously allocate scarce healthcare resources in the face of rising healthcare costs. In addition, the decision to concentrate on the most vulnerable groups is informed by the fact while most cases of ROP resolve spontaneously, severe untreated ROP is usually associated with poor ocular outcome. The large study population is an advantage with respect to the power of the study. Furthermore, the use of standardized protocols in screening, classification, and diagnosis of ROP and maternal diabetes reduces information bias.

Our study has several limitations. The retrospective single-center study design limits the external validity of the study, and hence its applicability to different settings. The study population was limited to infants with BW≤1500g which signifies that very preterm babies with BW>1500g were excluded from the study, especially those born at GA ≤30 weeks. Since the relationship between prematurity and ROP is widely acknowledged, this implies that the incidence of ROP and severe ROP may have been underrepresented in this study. The study was carried out on liveborn babies, and neonates who died prior to discharge were excluded from the study suggesting a potential bias. Lack on information on transferred babies may have impacted the result of the study by possibly underrepresenting the incidence of ROP, MDM, and other comorbidities. Since, most of the transfers were usually due to need for a higher level of care, this cohort would likely be sicker and potentially of lower gestational age and birth weight. Given that maternal diabetes is associated with higher incidence of intrauterine and neonatal deaths, the association between severe ROP and maternal diabetes may have been attenuated. Another limitation is that data on the degree of maternal glycemic control and duration of diabetes were not comprehensively collected and analyzed in the study.

## Conclusion

Maternal diabetes is an independent risk factor for severe ROP and the strength of association between maternal DM and ROP increased with increasing severity of ROP. Other factors positively associated with severe ROP were neonatal sepsis, neonatal steroid use and maternal chorioamnionitis, while prenatal steroid use was associated with reduced risk of severe ROP. Further studies are needed to understand the impact of the duration of diabetes as well as maternal glycemic control on the relationship between severe ROP and maternal diabetes.

## References

[1] Blencowe H, Lawn JE, Vazquez T, Fielder A, Gilbert C. Preterm-associated visual impairment and estimates of retinopathy of prematurity at regional and global levels for 2010. Pediatr. Res. 2013; 74(suppl. 1): 35–49. 10.1038/pr.2013.205 24366462 PMC3873709

[2] Quinn GE, Ying G, Bell EF, Donohue PK, Morrison D, Tomlinson AF, et al Incidence and early course of retinopathy of prematurity secondary analysis of the postnatal growth and retinopathy of prematurity (G-ROP) study. JAMA Ophthalmol. 2018; 136(12): 1383–1389. 10.1001/jamaophthalmol.2018.4290 30326046 PMC6583045

[3] Good WV, Hardy RJ, Dobson V, Palmer EA, Phelps DL, Quintos M, et al The incidence and course of retinopathy of prematurity: findings from the early treatment for retinopathy of prematurity study. Pediatrics. 2005; 116:15 10.1542/peds.2004-1413 15995025

[4] Darlow BA, Hutchinson JL, Henderson-Smart DJ, Donohue DA, Simpson JM, Evans NJ. Prenatal risk factors for severe retinopathy of prematurity among very preterm infants of the Australian and New Zealand Neonatal Network. Pediatrics. 2005; 115:990 10.1542/peds.2004-1309 15805375

[5] Early Treatment for Retinopathy of Prematurity Cooperative Group. Revised indications for the treatment of retinopathy of prematurity: results of the early treatment for retinopathy of prematurity randomized trial. Arch Ophthalmol. 2003; 121(12):1684–1694. 10.1001/archopht.121.12.1684 14662586

[6] Gilbert C. Retinopathy of prematurity: a global perspective of the epidemics, population of babies at risk and implications for control. Early Human Development. 2008; 84: 77–82 10.1016/j.earlhumdev.2007.11.009 18234457

[7] Fierson WM; American Academy of Pediatrics Section on Ophthalmology; American Academy of Ophthalmology; American Association for Pediatric Ophthalmology and Strabismus; American Association of Certified Orthoptists. Screening examination of premature infants for retinopathy of prematurity. Pediatrics. 2018; 142(6): e20183061 10.1542/peds.2018-3061 30478242

[8] Terry TL. Retrolental fibroplasia in the premature infant: V. further studies on fibroplastic overgrowth of the persistent tunica vasculosa lentis. Trans Am Ophthalmol Soc. 1944; 42:383–396 16693360 PMC1315141

[9] Tunay ZO, Ozdemir O, Acar DE, Oztuna D, Uras N. Maternal Diabetes as an independent risk factor for retinopathy of prematurity in infants with birth weight of 1500 g or more. Am J Ophthalmol. 2016; 168:201–206. 10.1016/j.ajo.2016.05.022 27287819

[10] Blanco CL, Baillargeon JG, Morrison RL, Gong AK. Hyperglycemia in extremely low birth weight infants in a predominantly Hispanic population and related morbidities. J Perinatol. 2006; 26:737–741 17. 10.1038/sj.jp.7211594 16929343

[11] Garg R, Agthe AG, Donohue PK, Lehmann CU. Hyperglycemia and retinopathy of prematurity in very low birth weight infants. J Perinatol. 2003; 23:186–194 10.1038/sj.jp.7210879 12732854

[12] Persson M, Shah PS, Rusconi F, Reichman B, Modi N, Kusuda S, et al Association of maternal diabetes with neonatal outcomes of very preterm and very low-birth-weight infants: an international cohort study. JAMA Pediatr. 2018; 172(9): 867–875. 10.1001/jamapediatrics.2018.1811 29971428 PMC6143059

[13] Lee JH, Hornik CP, Testoni D, Laughon MM, Cotten CM, Maldonado RS, et al Insulin, hyperglycemia, and severe retinopathy of prematurity in extremely low-birth-weight infants. Amer J Perinatol. 2016; 33(04): 393–400. 10.1055/s-0035-1565999 26485249 PMC4794341

[14] Bental Y, Reichman B, Shiff Y, Weisbrod M, Boyko V, Lerner-Geva L, et al Impact of maternal diabetes mellitus on mortality and morbidity of preterm infants (24–33 weeks’ gestation). Pediatrics. 2011; 128: e848–e855 10.1542/peds.2010-3443 21930550

[15] International Committee for the Classification of Retinopathy of Prematurity. The International classification of retinopathy of prematurity revisited. Arch Ophthalmol. 2005; 123: 991–999 10.1001/archopht.123.7.991 16009843

[16] Kim SJ, Port AD, Swan R, Campbell JP, Chan RV, Chiang MF. Retinopathy of prematurity: a review of risk factors and their clinical significance. Survey of ophthalmology. 2018; 63: 618–637. 10.1016/j.survophthal.2018.04.002 29679617 PMC6089661

[17] Celebi AR, Petricli IS, Hekimoglu E, Demirel N, Bas AY. The incidence and risk factors of severe retinopathy of rrematurity in extremely low birth weight infants in Turkey. Med Sci Monit. 2014; 20: 1647–1653 10.12659/MSM.892262 25220443 PMC4172092

[18] Akkoyun I, Oto S, Yilmaz G, Gurakan B, Tarcan A, Anuk D, et al Risk factors in the development of mild and severe retinopathy of prematurity. Journal of American Association for Pediatric Ophthalmology and Strabismus. 2006; 10(5): 449–453 10.1016/j.jaapos.2006.05.007 17070481

[19] Kumar P, Sankar MJ, Deorari A, Azad R, Chandra P, Agarwal R, et al Risk factors for severe retinopathy of prematurity in preterm low birth weight neonates. Indian J Pediatr. 2011; 78(7): 812–816 10.1007/s12098-011-0363-7 21340729

[20] Karna P, Muttineni J, Angell L, Karmaus W. Retinopathy of prematurity and risk factors: a prospective cohort study. BMC Pediatrics. 2005; 5: 18–26 10.1186/1471-2431-5-18 15985170 PMC1175091

[21] Mutlu FM, Altinsoy HI, Mumcuoglu T, Kerimoglu H, Kilic S, Kul M, et al Screening for retinopathy of prematurity in a tertiary care newborn unit in Turkey: frequency, outcomes, and risk factor analysis. J Pediatr Ophthalmol Strabismus. 2008; 45(5): 291–298. 10.3928/01913913-20080901-12 18825902

[22] Bas AY, Demirel N, Koc E, Isik DU, Hirfanoglu IM, Tunc T. Incidence, risk factors and severity of retinopathy of prematurity in Turkey (TR-ROP study): a prospective, multicentre study in 69 neonatal intensive care units. Br J Ophthalmol. 2018; 0: 1–6. 10.1136/bjophthalmol-2017-311789 PMC6287567 29519879

[23] Yau G, Lee J, Tam V, Liu C, Wong I. Risk factors for retinopathy of prematurity in extremely preterm Chinese infants. Medicine. 2014; 93 (28): 314–320 10.1097/MD.0000000000000314 PMC4603108 25526484

[24] Holmstrom G, Thomasson P, Broberger U. Maternal risk factors for retinopathy of prematurity—a population-based study. Acta Obstet Gynecol Scand. 1996; 75: 628–635 10.3109/00016349609054687 8822655

[25] Wei J, Sung F, Lee C, Chang C, Lin R, Lin C, et al Low birth weight and high birth weight infants are both at an increased risk to have type 2 diabetes among schoolchildren in Taiwan. Diabetes Care. 2003; 26:343–348 10.2337/diacare.26.2.343 12547860

[26] Purohit DM, Ellison RC, Zierler S, Miettinen OS, Nadas AS. Risk factors for retrolental fibroplasia: experience with 3,025 premature infants. National collaborative study on patent ductus arteriosus in premature infants. Pediatrics. 1985; 76: 339–344 2863804

[27] Kaempf JW, Kaempf AJ, Wu Y, Stawarz M, Niemeyer J, Grunkemeier G. Hyperglycemia, insulin and slower growth velocity may increase the risk of retinopathy of prematurity. J Perinatol. 2011; 31(4): 251–257. 10.1038/jp.2010.152 21233796

[28] Rehan V, Moddemann D, Casiro O. Outcome of very-low-birth-weight (<1,500 Grams) infants born to mothers with diabetes. Clin Pediatr. 2002; 41: 481–491 10.1177/000992280204100705 12365310

[29] Early Treatment for Retinopathy of Prematurity Cooperative Group. The incidence and course of retinopathy of prematurity: findings from the early treatment for retinopathy of prematurity study. Pediatrics. 2005; 116: 15–23 10.1542/peds.2004-1413 15995025

[30] Palmer EA, Flynn JT, Hardy RJ, Phelps DL, Phillips CL, Schaffer DB, et al Incidence and early course of retinopathy of prematurity. Ophthalmology. 1991; 98: 1628–1640 10.1016/s0161-6420(91)32074-8 1800923

[31] Movsas TZ, Spitzer AR, Gewolb IH. Postnatal corticosteroids and risk of retinopathy of prematurity. J Aapos. 2016; 20(4): 348–352 10.1016/j.jaapos.2016.05.008 27318211

[32] Mitra S, Aune D, Speer CP, Saugstad OD. Chorioamnionitis as a risk factor for retinopathy of prematurity: a systematic review and meta-analysis. Neonatology. 2014; 105(3): 189–199 10.1159/000357556 24481268

[33] Console V, Gagliardi L, De Giorgi A, De Ponti E. Retinopathy of prematurity and antenatal corticosteroids. The Italian ROP Study Group. Acta Biomed Ateneo Parmense. 1997; 68 Suppl. 1: 75–79 10021720

